# Identification of Small Molecule Inhibitors of Human Cytomegalovirus pUL89 Endonuclease Using Integrated Computational Approaches

**DOI:** 10.3390/molecules28093938

**Published:** 2023-05-07

**Authors:** Mazen Almehmadi, Ihtisham Ul Haq, Ahad Amer Alsaiari, Fahad M. Alshabrmi, Osama Abdulaziz, Mamdouh Allahyani, Mohammed Aladhadh, Alaa Shafie, Abdulelah Aljuaid, Rema Turki Alotaibi, Jawad Ullah, Nada Saud Alharthi

**Affiliations:** 1 Department of Clinical Laboratory Sciences, College of Applied Medical Sciences, Taif University, Taif 21944, Saudi Arabia; 2 Department of Physical Chemistry and Technology of Polymers, Silesian University of Technology, M. Strzody 9, 44-100 Gliwice, Poland; 3 Joint Doctoral School, Silesian University of Technology, Akademicka 2A, 44-100 Gliwice, Poland; 4 Department of Medical Laboratories, College of Applied Medical Sciences, Qassim University, Buraydah 51452, Saudi Arabia; 5 Department of Food Science and Human Nutrition, College of Agriculture and Veterinary Medicine, Qassim University, Buraydah 51452, Saudi Arabia; 6 Department of Chemistry, Hazara University, Mansehra 21120, Pakistan

**Keywords:** pUL89, cytomegalovirus, virtual screening, molecular docking, MD simulation

## Abstract

Replication of Human Cytomegalovirus (HCMV) requires the presence of a metal-dependent endonuclease at the C-terminus of pUL89, in order to properly pack and cleave the viral genome. Therefore, pUL89 is an attractive target to design anti-CMV intervention. Herein, we used integrated structure-based and ligand-based virtual screening approaches in combination with MD simulation for the identification of potential metal binding small molecule antagonist of pUL89. In this regard, the essential chemical features needed for the inhibition of pUL89 endonuclease domain were defined and used as a 3D query to search chemical compounds from ZINC and ChEMBL database. Thereafter, the molecular docking and ligand-based shape screening were used to narrow down the compounds based on previously identified pUL89 antagonists. The selected virtual hits were further subjected to MD simulation to determine the intrinsic and ligand-induced flexibility of pUL89. The predicted binding modes showed that the compounds reside well in the binding site of endonuclease domain by chelating with the metal ions and crucial residues. Taken in concert, the in silico investigation led to the identification of potential pUL89 antagonists. This study provided promising starting point for further in vitro and in vivo studies.

## 1. Introduction

At an alarmingly high rate of prevalence all over the world, human Cytomegalovirus or HCMV is one of the most widespread herpesviruses [1]. The prevalence of HCMV infection in the human population varies widely, with seroprevalence rates ranging from 30% to 90% in developed countries. The prevalence of HCMV infection increases with age, reflecting the greater likelihood of exposure over time [2]. Human cytomegalovirus is typically benign in healthy individuals but can cause serious complications in fetuses, neonates, and immunocompromised patients. In these populations, HCMV infection can lead to a range of damaging effects, including birth defects, developmental delays, and life-threatening conditions such as encephalitis, pneumonia, and hepatitis [3,4,5]. So far infected individuals are treated palliatively with approved HCMV drugs such as an antisense 21 mer phosphorothioate oligonucleotide, Fomivirsen, DNA polymerase inhibitors including; Ganciclovir, Valganciclovir, Cidofovir, and Foscarnet and a DNA terminase inhibitor, Letermovir to alleviate infection [6,7,8,9,10,11]. All these antiviral drugs are associated with a number of side effects [12,13]. Moreover, many viral strains have emerged with resistance to these antiviral drugs [14]. There is an urgent need for the development of novel antiviral therapies with good pharmacological profile and reduced side-effects. Over the past two decades, efforts have been made to design new chemical compounds as inhibitors of CMV with potent antiviral activity [15,16,17,18,19,20,21,22,23]. Despite of such extensive research, no pUL89 therapeutic reached the clinical development and optimization.

HCMV terminase is a protein composed of two subunits, a small and a large subunit. The small subunit binds the DNA, while the large subunit has both endonuclease and ATPase activities. The endonuclease activity cleaves the DNA, and the ATPase activity mediates the translocation of the DNA into the preformed capsid. This process helps in the formation of unit-length genomes during packaging, which is crucial for the maturation of the HCMV virus. Therefore, among many therapeutic targets, CMV terminase machinery, is an attractive therapeutic target to developed anti-CMV therapies [24]. It has been also suggested that blocking or targeting CMV terminase complex with terminase inhibitors may have less undesired effects than targeting the other CMV proteins. HCMV terminase complex consist of three subunits namely, UL56, UL89, and UL51 [25]. UL56, is thought to play multiple roles in the packaging process: (i) mediating specific binding to pac motifs on the concatamers, (ii) providing energy for DNA translocation to the procapsids, and (iii) associating with the capsid to enable DNA entry. On the other hand, pUL89, primarily plays a role in cleaving the concatemeric DNA into unit-length genomes. The UL89 protein (pUL89) consists of N-terminal and C-terminal domains. The N-terminal domain consists of 1-417 amino acids with adenosinetriphosphatase activity while C-terminal consists of 418-674 amino acids with nuclease activity [26]. Developing specific inhibitors for viral UL89-N may be difficult due to the highly conserved ATP binding motif in a wide variety of proteins. Instead, developing HCMV inhibitors that can specifically target the nuclease activity of UL89-C is a more effective approach. In this regard researchers in academia have discovered several potential inhibitors of pUL89 with low micromolar biological activity. In 2017, Wang and coworkers discovered hydroxypyridonecarboxylic acid analogs as inhibitors of pUL89 endonuclease activity using ELISA format as a screening assay [27]. The year later, the same group performed the structure-activity relationship of the hydroxypyridonecarboxylic acid scaffold to under-stand the impact of the carboxylic acid functionality and the N1 site on the activity of pUL89 [28]. Their efforts led to the identification of several analogues that effectively inhibit both pUL89-C and HCMV with low micromolar potency and no observed cytotoxicity at concentrations up to 200 μm. Several similar studies were also conducted that led to the identification of potential pUL89 inhibitors with different chemical scaffold [22,29].

In this study, we identified potential virtual hits against pUL89 C-terminal domain of HCMV by using computational techniques. To utilized the available chemical and biological information of pUL89 inhibition we integrated the computational structure and ligand-based drug design approaches. In the result of multistep screening we identified potential chemical compounds having diverse scaffold from the known antagonists of pUL89. The findings of our study could be further used for in vitro experimental studies to validate the potencies of the identified virtual hits against CMV.

## 2. Results and Discussion

Several pUL89 CMV terminase subunit antagonists have been documented/discovered using experimental methods. This study aims to discovered potential lead compounds against pUL89 by using computational approaches to hasten the drug design and development process. A mounting body of evidences suggested that integrated structure and ligand-based virtual screening is a more effective tool that has the potential to surpass a single method due to the utilization of chemical and biological information [30,31,32]. Herein, we integrated structure and ligand-based drug design approaches to identify potential lead compounds against pUL89 of CMV.

### 2.1. Structure and Ligand-Based Virtual Screening

**Figure 1:**
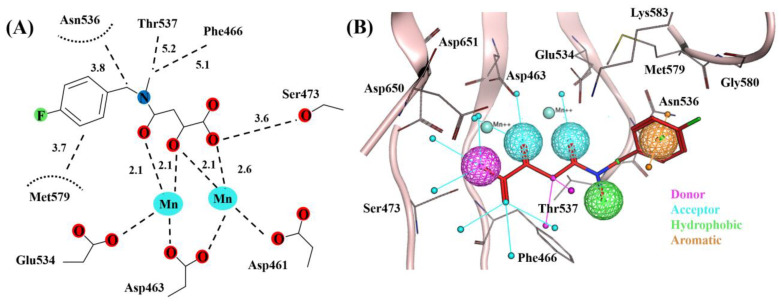
Binding mode of cognate ligand in PDB ID 6EY7 within the binding site of pUL89. (**A**) 2D interaction network between the ligand and protein (adapted from Ref. [29]). (**B**) Essential chemical features including, hydrogen bond donor (magenta), hydrogen bond acceptor (cyan), hydrophobic (green) and aromatic (orange), of cognate ligand required for the inhibition of pUL89-C nuclease activity.

The X-ray crystal structure of C-terminal domain of pUL89 in complex with α,γ-diketoacid analogue inhibitor, determined by Bongarzone and co-workers at resolution of 2.90 Å provided detail information to drive structure-based drug design campaign against CMV [29]. We begin this study by utilizing the details of molecular interactions between pUL89 and 2 (cocrystallized ligand in PDB 6EY7). The crystal structure of 2 in complex with pUL89 suggested that α,γ-diketoacid core chelate with the two Mn^2+^ ions (Figure 1A). The three oxygen atom of α,γ-diketoacid chelated with the Mn^2+^ ions, with the central oxygen atom serving as a bridge between the ions. Similarly, the 4-fluorophenyl ring move towards the hydrophobic cleft made by Asn536, Thr537 and Met579 (Figure 1A). Moreover, Bongarzone and coworkers suggested that to increase the hydrophobic contact with the nearby Phe466, the methyl group of 2 could be replaced with a bulky alkyl. By utilizing this information, we selected five essential chemical feature including, two hydrogen bond acceptor, one hydrogen bond donor; to interact with two Mn^2+^ ions, one aromatic and one hydrophobic feature to interact with Asn536, Thr537, Met579 and Phe466 (Figure 1B).

The selected chemical features were further utilized to screen the ZINC and ChEMBL database to extract the compounds with the similar features. On the basis of chemical feature-based screening we obtained ~400 potential hit compounds against CMV. The obtained compounds were subjected to the molecular docking studies to delineate the binding mode and binding affinity with pUL89. All the compounds showed the binding affinity in the range of −7.75 to −3.32 with significant interactions with the crucial residues and metal ions. Further, the compounds were selected based on the docking score of cognate ligand (−5.31 kcal/mol). Therefore, a total of 136 compounds that scored higher than −5.31 in the docking analysis were chosen for further analysis. To further, extract the potential hit compounds, the ligand shape-based similarity filter was employed. In this connection, two known inhibitors of CMV pUL89 with low micromolar IC_50_ were selected [29]. All the docked compounds were subjected to shape similarity filter using MOE with the threshold of 0.75. In the result of screening, 28 compounds were obtained with the tanimoto similarity coefficient >0.75. In the final step of the screening, the obtained 28 compounds were visually analysed for intramolecular interactions with the crucial residues, metal chelation and complementarity between ligand and the binding site of the pUL89. Among the analysed compounds, seven compounds (six compounds from ChEMBL database and one compound from ZINC database) were found to fulfil the criteria of visual inspection. The structures, binding affinity and molecular interactions between ligands and the crucial residues and tanimoto similarity with 2 are presented in Table S1. Further, three compounds were selected on the basis of binding scores, interactions and tanimoto similarity and subjected to MD simulation. From ChEMBL database SK-1, SK-2, SK-3, SK-4, SK-5 and SK-6 showed the highest binding score than the 2, cognate ligand (Table S1). However, SK-1 and SK-2 showed more significant interactions thus these two compounds were selected from ChEMBL database for MD simulation (Table S1). Similarly, from ZINC database only SK-7 was obtained from shape similarity filter which also showed good binding score and significant interactions with the crucial residues, thus it was selected for MD simulation. Figure S1 illustrates the graphical representation of the virtual screening workflow.

### 2.2. Molecular Dynamics Simulation

MD simulation is a state-of-the-art simulation method for studying the physical motion of target protein in the presence of ligands along with the various interactions within a system [33,34,35]. Herein, three replicas of 100 ns simulations were carried out for Apo pUL89 both in its free form and in complex with SK-1, SK-2, and SK-7, in order to evaluate the protein’s motions and flexibility, which contribute to the interaction dynamics of protein-ligand complexes. The results of the first replica are presented here, while the graphs for the second and third replicas are provided in Figures S2–S5. The protein-ligand complexes intrinsic and ligand-induced flexibility was evaluated by plotting root mean square deviation (RMSD), root mean square fluctuation (RMSF) and Radius of Gyration (Rg).

**Figure 2:**
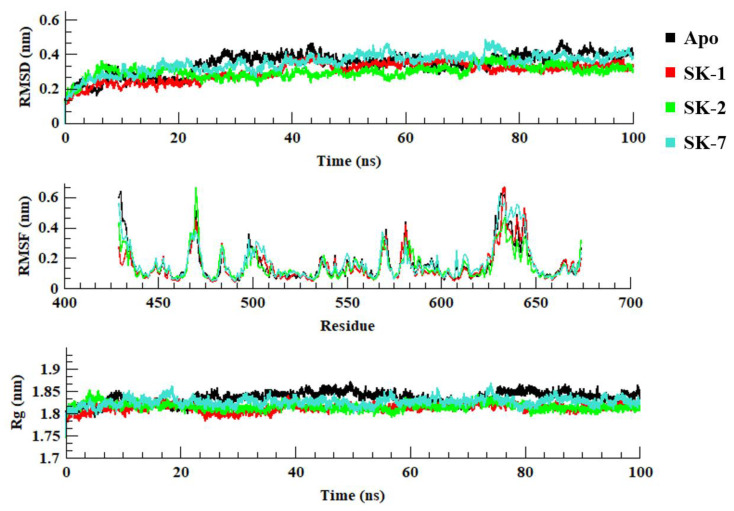
Root mean square deviation, root mean square fluctuation and radius of gyration of Apo pUL89 and in complex with three virtual hits during the course of simulation.

**Figure 3:**
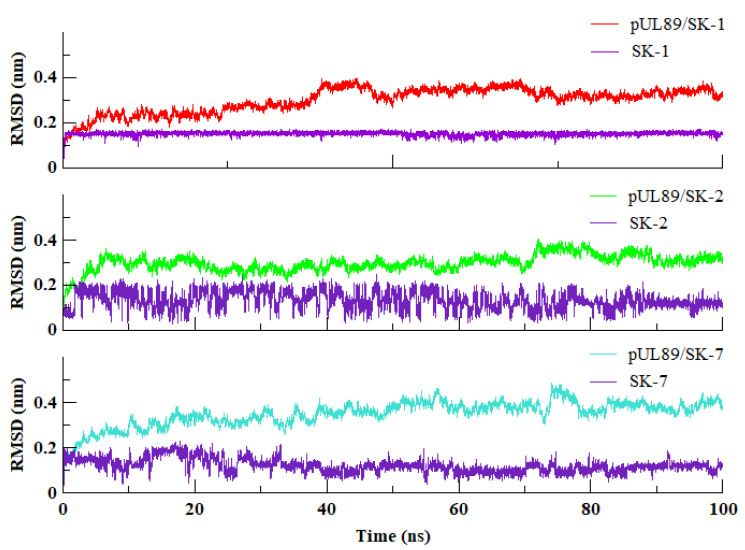
Root mean square deviation, backbone carbon atom of pUL89 complexes and ligands during the course of simulation.

The root-mean-square deviation (RMSD) measures the difference between the positions of atoms in the target protein during a simulation and the positions in the initial reference structure. A low RMSD value indicates that the system is stable and has reached convergence, while larger fluctuations may indicate that the system is not yet fully equilibrated. The stability of simulated systems was evaluated by RMSD of backbone carbon atoms (Figure 2). The RMSD plot of Apo and protein-ligand complexes demonstrated the significant stability for all the simulated systems. All the systems were fluctuated between 0.15 nm to 0.45 nm with variable fluctuation. Upon closer examine it was observed that SK-1 and SK-2 significantly stabilized the endonuclease domain of pUL89 upon binding in comparison of SK-7. Visual analysis of the trajectories revealed that, during the simulations, the ligands remained within the protein pocket and experienced less fluctuation compared to their initial placement as depicted in Figure 3. SK-1 showed the most stable RMSD with no major fluctuations. Whereas, SK-2 was relatively unstable, however the deviations were in the acceptable range (0–0.2 nm). Similarly, SK-7 also showed the relatively stable RMSD throughout the course of the simulation. This observation delineated that the selected compounds were reside well in the binding site of pUL89 throughout the 100 ns of simulation.

Root-mean-square fluctuations (RMSF) analysis is a method used to calculate the time-dependent fluctuations of each residue in a protein, which provides information about its flexibility. The RMSF values can be used to identify regions of the protein that are more flexible or rigid, which can be important for understanding its function and stability. Similarly, to study the ligand-induced flexibility/rigidity of protein residues, RMSF was calculated (Figure 2). RMSF plot of all the simulated systems showed the variable fluctuations of amino acid residues with almost similar magnitude. The terminal helix region (from Leu627 to Val656) was most fluctuated in all the systems. However, the crucial residues including Asn536, Thr537, and Met579 were found to be rigid throughout the whole simulation. However, Phe466 was found to be highly fluctuated in all systems in comparison of other crucial residues.

The radius of gyration (Rg) is used to assess the compactness of protein structure. Similarly, to evaluate the compactness or folding and unfolding of pUL89 upon binding of virtual hits, radius of gyration was plotted (Figure 2). High Rg values are indicative of a less compact (more unfolded) structure due to high conformational entropy, whereas low Rg values indicate a more compact and more stable structure (more folded). All the systems projected the Rg score between 1.75 nm to 1.85 nm. As expected, the protein-ligand complexes were more compact in comparison of Apo system which indicated that the binding of virtual hits significantly compact the pUL89.

**Figure 4:**
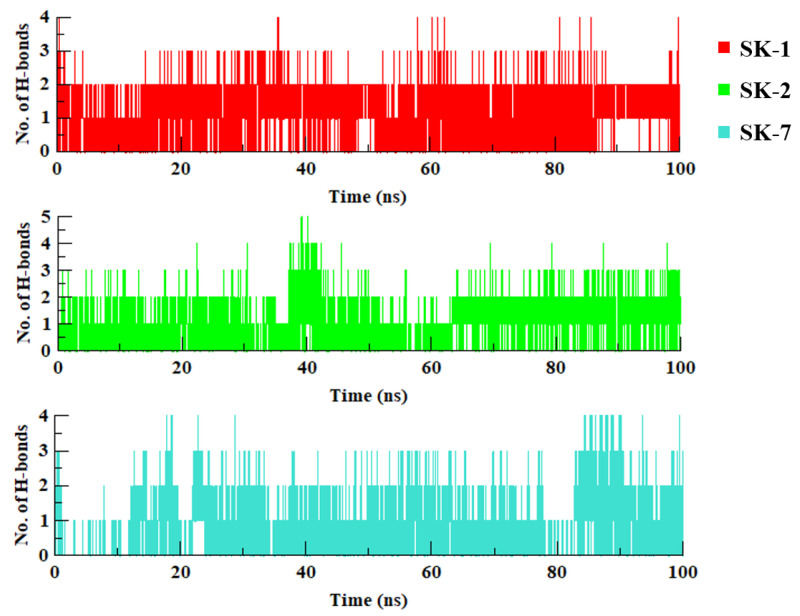
The number of hydrogen bonds mediated by selected virtual hits during the course of simulation.

Similarly, to evaluate the intramolecular hydrogen bonds between the selected virtual hits and pUL89 throughout the simulation, the hydrogen bonds were calculated using hbond module of Gromacs. As evident from Figure 4, all the compounds maintain two hydrogen bonds with significant occupancy. Further, to appraise the other intramolecular interactions with the crucial residues and metal ions, the simulated trajectories were visually analysed using VMD [36].

**Figure 5:**
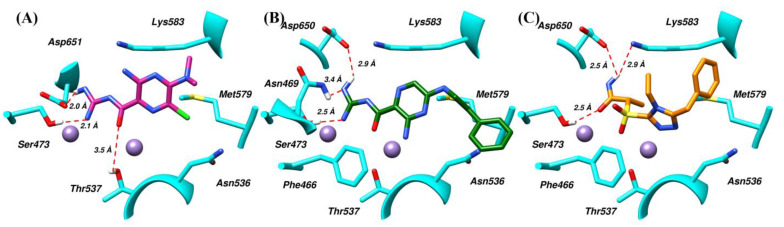
Binding modes of virtual hits. The compound (**A**) SK-1, (**B**) SK-2 and (**C**) SK-7 and the pUL89-C residues surrounded by the selected virtual hits are shown in stick representation. The two Mn^2+^ ions are shown in plum colour and red dotted lines represented the hydrogen bonds.

Upon examination of simulated trajectories of SK-1, SK-2 and SK-5, it was observed that most of the docking interactions were persist and all the compounds were positioned at the binding site throughout the course of 100 ns. Phe466 Asn469, Ser473, Asn536, Thr537, and Met579 majorly contributed to the binding of ligands (Figure 5). In case of SK-1, carboxamide and carbamimidoyl groups of the compound chelate with the metal ions (Figure 5A). In addition, the terminal amino group of carbamimidoyl moiety was observed to mediate hydrogen bonds with Ser473 and Asp651. Similarly, the carbonyl oxygen of carboxamide group establishes a hydrogen bond with Thr537. Further, the protein-ligand complex was stabilized by a network of hydrophobic interactions mediated by Asn536, Met579 and Lys583. In case of SK-2, similar to SK-1 its carbamimidoyl moiety mediate three hydrogen bonds with Asn469, Ser473, and Asp650 (Figure 5B). In addition, amino group substituted on pyrazine ring mediate hydrogen bond with Asn536. Moreover, metal chelation was also observed with carboxamide and carbamimidoyl group of SK-2. Further, Phe446 mediate arene-H interaction and significantly contributed in the stabilization of the complex. Similarly, Asn536, Thr537, Met579 and Lys583 interacted with the ligand by mediating hydrophobic interactions. In case of SK-7, the oxygen of amide and sulfonyl group serve as a metal sequester (Figure 5C). The ligand also mediates three hydrogen bonds with Ser473, Lys583 and Asp650. Whereas, Phe466 stacked against the triazole ring by mediating π-π interaction. Further, a network of hydrophobic interactions was observed with Asn536, Thr537, Met579 and Lys583. Interestingly, most of the hydrophobic interactions were persist throughout the MD simulation, however, the occupancy of hydrogen bonds was lesser than the other interactions as depicted in Table 1. All the compounds showed similar type of interactions like the cognate ligand (Figure 1A) which may attribute to the potency of these compounds.

### 2.3. Binding Free Energy

**Figure 6:**
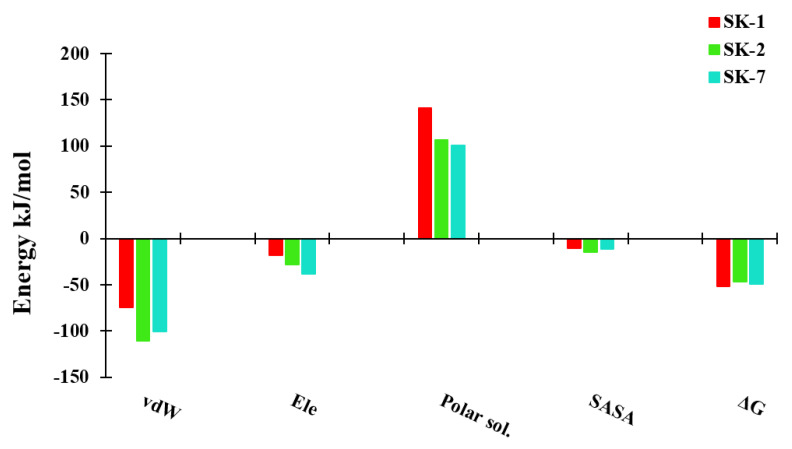
The relative binding free energy of three simulated complexes with the contribution of individual energy terms.

The relative binding free energy of three complexes were predicted by MMGBSA approach. The predicted binding affinities of pUL89/SK-1, pUL89/SK-2 and pUL-89/SK-7 were found to be −51.99 kJ/mol, −46.63 kJ/mol and −48.99 kJ/mol, respectively. As expected SK-1 complex showed the highest binding affinity followed by SK-7 and SK-2 (Figure 6). Further to evaluate the contribution of energy term in the binding of ligands, the binding free energy was decomposed into individual energy term. It was observed that van der Waal and electrostatic energies contributed highly in the binding of ligands. As discussed in Section 2.2, non-polar and aromatic chemical groups present in the compounds established a network of hydrophobic interactions with Phe466, Asn536, Thr537, Met579, and Lys583. These interactions can be primarily attributed to significant van der Waals interactions. Additionally, the presence of polar groups in the compound facilitated hydrogen bond contacts, which favorably contributed to the electrostatic interactions in the ligand binding process. Moreover, the polar solvation energy contributed unfavorably in the binding process. The binding free energy calculations were consistent with the MD simulation results. However, they did not agree well with the docking score, as the compound with the highest docking score did not exhibit the strongest binding energy (Table 2). This discrepancy may be attributed to the fact that the docking score is a static measure that does not consider the time-dependent dynamics of protein-ligand complexes. Therefore, the MD simulation results were considered for evaluating the binding energy of the ligands.

## 3. Materials and Methods

### 3.1. Database Collection and Preparation

To identify the small molecule inhibitors of pUL89 of CMV, two commercially available databases of chemical compounds with drug-like properties were utilized. A “drug-like” subset of ZINC database consists of ~0.4 million compounds was downloaded [37]. Similarly, a screening dataset of ~0.3 million chemical compounds from ChEMBL database (European Bioinformatics Institute, Cambridge, United Kingdom) was created by applying filters including small molecules, rule of five and molecular weight of 200–700 g/mol [38]. The collected compounds (~0.7 million) were converted into 3D format by using OpenEye scientific software 2.3.1 (Cadence Molecular Sciences, NM, USA). Subsequently, the compounds were optimized and minimized by using MMFF94 force field.

### 3.2. Target Protein Preparation

CMV terminase subunit UL89 in complex with α,γ-diketoacid analogue inhibitor and two Mn^2+^ ions was retrieved from Protein DataBank under the accession code of 6EY7 [29]. Since there were no conserved water molecules reported, all the water molecules were deleted. The crystal structure was subjected to geometry correction process which entails assigning the correct bond order, terminal capping and addition of missing atoms followed by protonation. Moreover, the missing loops were modeled using the Loop Modeler module of MOE v.2019.01 (Chemical Computing Group, QC, Canada). Afterwards, the energy minimization was carried out by using AMBER10:EHT force field. Finally, the structure was saved into pdb format for further processing.

### 3.3. Structure and Ligand-Based Virtual Screening

Herein, structure and ligand-based virtual screening approaches were integrated to utilize the available chemical and biological information for the identification of potential pUL89 inhibitors. Initially, the molecular interactions between pUL89 and cocrystallized ligand were taken into consideration and essential chemical features were defined [29]. The essential features included two hydrogen bond acceptor, one hydrogen bond donor; to interact with two Mn^2+^ ions and Ser473, one aromatic and one hydrophobic feature to interact with Asn536, Thr537, Met579 and Phe466. The five selected features were defined as 3D query to search potential virtual hits with the similar chemical features from ZINC and ChEMBL databases.

In the first step all the prepared ~0.7 million compounds were screened against the defined query. In the second step, the resultant ~400 virtual hits were subjected to molecular docking-based screening. The crystal structure of UL89 (PDB ID 6EY7) was utilized for docking simulation. The docking grid was defined by selecting the atomic coordinates of the cognate ligand and the obtained virtual hits in the first steps were docked into the defined site. The Triangular Matcher was used as a placement method with induce fit docking protocol. For each compound maximum of ten conformations were generated and five best were retained. The resultant poses were scored using London dG and GBVI/WSA dG as scoring and rescoring function, respectively. Afterwards, the compounds were shortlisted based on the docked score of cognate ligand and as a result 136 compounds were selected for further analysis. In the next step, the top ranked compounds were prioritized based on the shape-similarity of pUL89 known inhibitors using the TGD (topological and geometrical descriptors) based on its ability to effectively capture the structural diversity and complexity of chemical compounds. The obtained virtual hits were searched for the similar shape with that of selected known inhibitors. As a result, 28 compounds were filter out with the Tanimoto similarity coefficient >0.75. In the final step, the compounds were visually analysed for hydrogen bonding and hydrophobic interactions with the crucial residues, Mn^2+^ chelation and complementarity between ligand and the binding site of the pUL89. Finally, the three compounds were selected subjected to molecular dynamics simulation.

### 3.4. Molecular Dynamics Simulation

To evaluate the intrinsic and ligand-induced flexibility of pUL89 terminase subunit of CMV, MD simulation was carried out using Gromacs v2021.1 using the Amber99 force field [39]. The topologies for the compounds were generated from Automated Topology Builder. The parameters for Mn^2+^ were obtained from http://amber.manchester.ac.uk/ (accessed on 24 January 2023) and converted into proper units compatible with Gromacs. The complex structures were placed in a cubic box of TIP3P water model. Further, the counter ions were added to neutralize the systems. The systems were energy minimized using the steepest descent algorithm with maximum force <1000 kJ mol^−1^ nm^−1^. Long-range electrostatics interactions were treated by using the Particle Mesh Ewald (PME) method, with a cutoff of 12 for van der Walls interactions and Coulomb interactions. To obtain further convergence, the systems were equilibrated in an NVT and NPT ensemble for 200 ps at temperature of 300 K and pressure of 1 bar using Berendsen thermostat. Finally, the well equilibrated systems were subjected to three replicas of 100 ns of final production run and energies and coordinates were saved at every 10 ps. The trajectories were analysed to determine the stability of the complexes by plotting root mean square deviation (RMSD), root mean square fluctuation (RMSF), Radius of Gyration (Rg) and intramolecular interactions between the target protein and the ligands.

### 3.5. Binding Free Energy Calculation

The relative binding free energies of the selected virtual hits pUL89 of CMV were predicted by using molecular mechanical-generalized Born surface area (MM-GBSA) approach. For binding free energy calculations of each system, snapshots were extracted from the last 5 ns of MD trajectories. The MM-GBSA approach was calculated using the g_mmpbsa tool of Gromacs [40]. The calculations were based on the following equations

ΔGbind = Gcomplex − Gprotein − Gligand    = ΔE_MM_ + ΔG_GB_ + ΔG_SA_ − TΔS        = ΔE_vdw_ + Δ_Eele_ + ΔG_GB_ + ΔG_SA_ − TΔS

where ΔE_MM_ represent the van der Waals energy contribution (ΔEvdw) and electrostatic energy contribution (ΔEele) of the gas-phase interaction energy between protein-ligand complex, ΔG_GB_ represents the polar while ΔG_SA_ represents the nonpolar components of desolvation free energy. −TΔS is the entropy contribution at temperature T. 

## 4. Conclusions

Cytomegalovirus (CMV) is a common virus that results in substantial morbidity and mortality in immunocompromised persons. The therapeutic management of CMV entails the use of antiviral drugs and vaccines. The current therapeutics are associated with a number of side effects. Moreover, many CMV strains are emerged that have developed resistance to these therapeutic interventions. Thus, there is a pressing need to develop potential drug candidates against CMV. In this study we identified potential lead compounds against pUL89 of HCMV with different chemical scaffold from the previously reported inhibitors. In the multistep virtual screening integrated with structure and ligand-based drug design approaches we determined seven virtual hits against the target protein from commercially available ZINC and ChEMBL database. Among these three compounds were further evaluated by MD simulation. The results demonstrated the significant stability of the target protein upon binding of the compounds with similar type of interactions like the previously known inhibitors with low micromolar activity. Overall, these in silico findings can be used for further in vitro studies to validate the antiviral potential of these compounds against CMV.

## Figures and Tables

**Table 1:** Residue-specific occupancy of hydrogen bonding and hydrophobic interactions during MD simulation.

System	Type of Interaction	Residue	Distance with Highest Occupancy (Å)	% Occupancy
SK-1	Hydrogen bonding	Ser473	2.5	63
		Thr537	3.5	69
		Asp651	2.5	78
	Hydrophobic	Asn536	5.0	35
		Met579	--	--
		Lys583	4.6	88
SK-2	Hydrogen bonding	Ser473	2.2	57
		Asn469	3.6	54
		Asp650	--	--
	Hydrophobic	Phe466	4.4	81
		Asn536	4.1	98
		Thr537	3.5	66
		Met579	5.2	84
SK-7	Hydrogen bonding	Ser473	3.5	51
		Lys583	3.1	76
		Asp650	2.3	80
	Hydrophobic	Phe466	4.4	74
		Asn536	4.1	62
		Thr537	--	--
		Met579	4.8	91
		Lys583	4.1	72

**Table 2:** Comprehensive detail of selected virtual hits against CMV including chemical structures, database IDs, docking scores and binding energy.

Code	Structure	Database ID	Binding Score (kcal/mol)	Binding Energy (kJ/mol)
SK-1	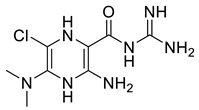	ChEMBL465179	−6.10	−51.99
SK-2	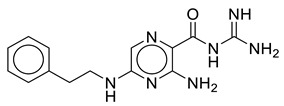	ChEMBL1962850	−6.15	−46.63
SK-7	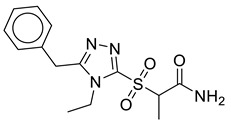	ZINC79951807	−6.33	−48.99

## Data Availability

Not applicable.

## Supplementary Materials

The following supporting information can be downloaded at: https://www.mdpi.com/article/10.3390/molecules28093938/s1, Figure S1: Workflow of virtual screening; Figure S2: RMSD, RMSF and Rg of three replicas of 100ns MD simulation for Apo pUL89; Figure S3: RMSD, RMSF and Rg of three replicas of 100ns MD simulation for pUL89/SK-1 complex; Figure S4 RMSD, RMSF and Rg of three replicas of 100ns MD simulation for pUL89/SK-2 complex; Figure S5: RMSD, RMSF and Rg of three replicas of 100ns MD simulation for pUL89/SK-7 complex; Table S1: Comprehensive detail of selected virtual hits against HCMV including chemical structures and results of docking studies.
