# Supplementary material for: Identification of Small Molecule Inhibitors of Human Cytomegalovirus pUL89 Endonuclease Using Integrated Computational Approaches
Source: Molecules. 2023 May 7;28(9):3938. doi: 10.3390/molecules28093938 (PMC10180037; doi:10.3390/molecules28093938)
Supplement: Supplementary file 1 [file molecules-28-03938-s001.zip › molecules-2307618-supplementary.pdf]

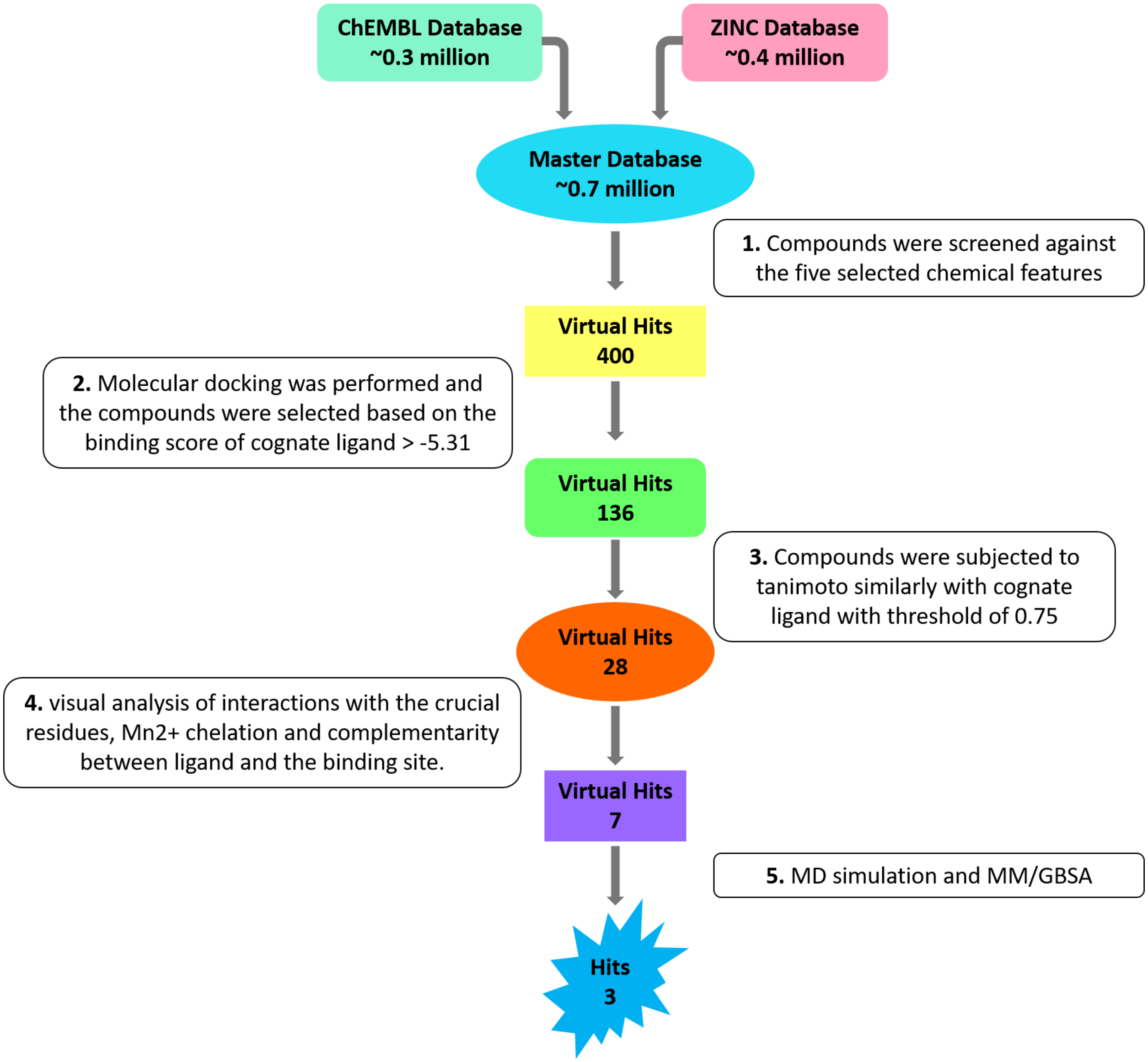

Figure S1. The schemaric represetation of structure and ligand-based virtual screeing.

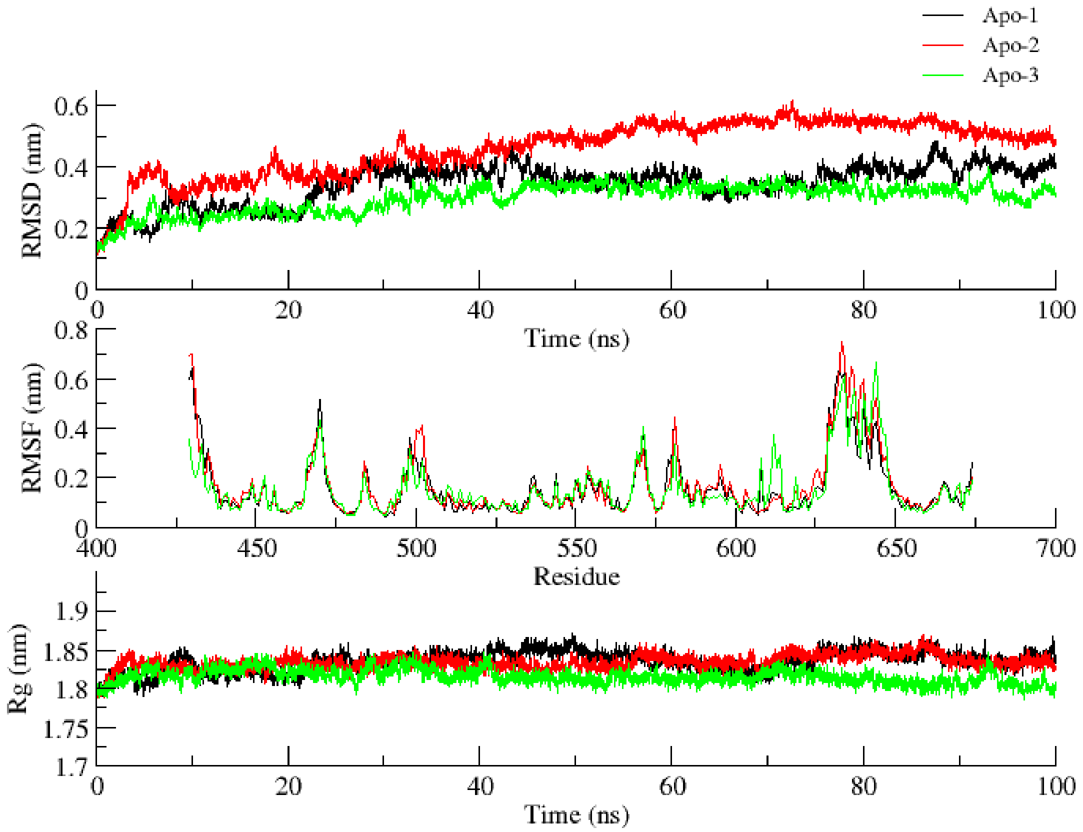

Figure S2. Root mean square deviation, root mean square fluctuation and radius of gyration of three replicas of 100ns MD simulation for Apo pUL89

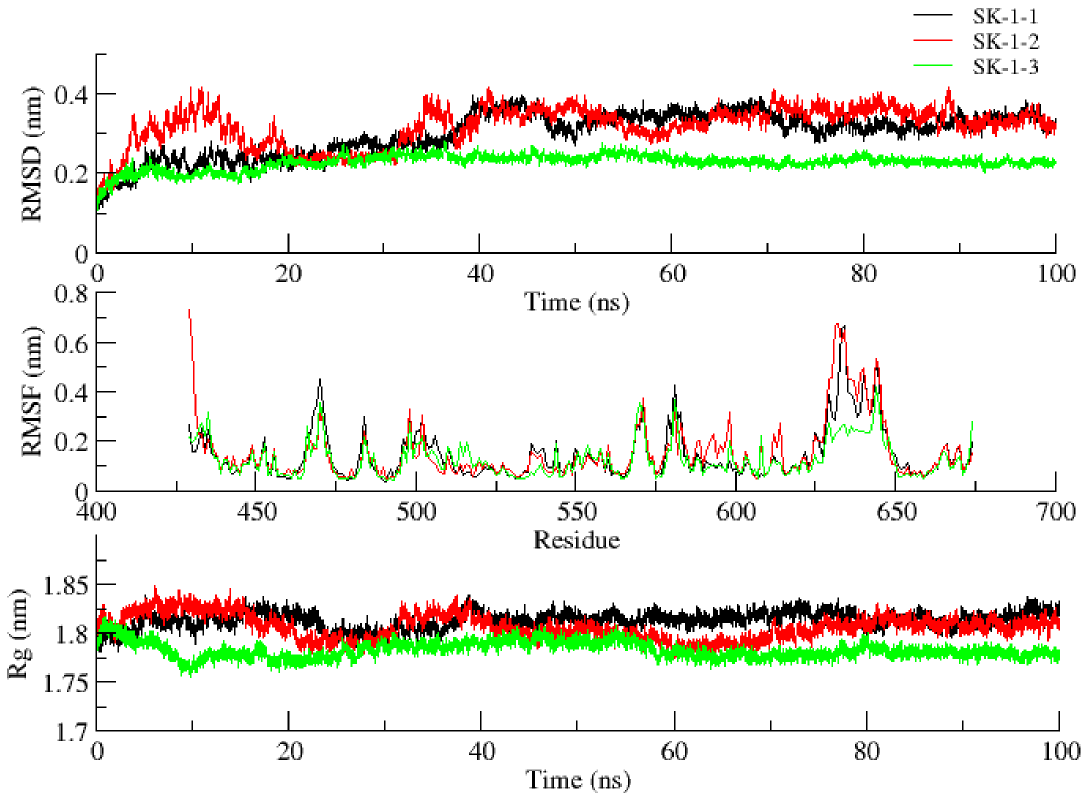

Figure S3. Root mean square deviation, root mean square fluctuation and radius of gyration of three replicas of 100ns MD simulation for pUL89/SK-1 complex

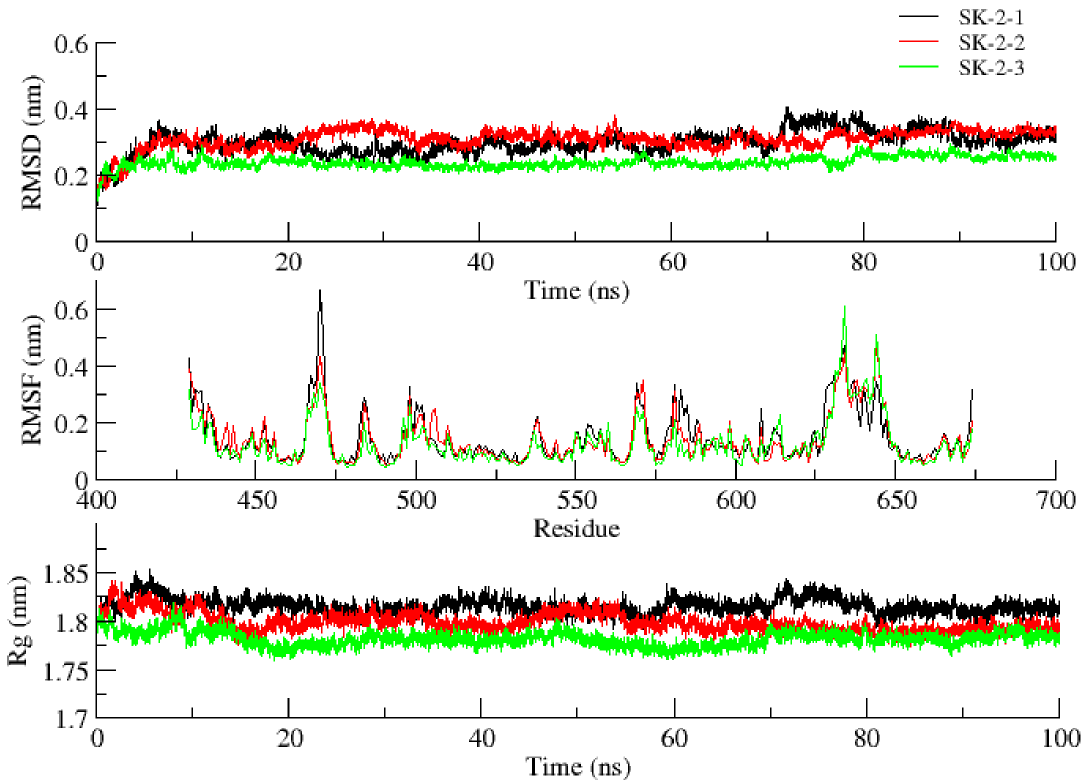

Figure S4. Root mean square deviation, root mean square fluctuation and radius of gyration of three replicas of 100ns MD simulation for pUL89/SK-2 complex

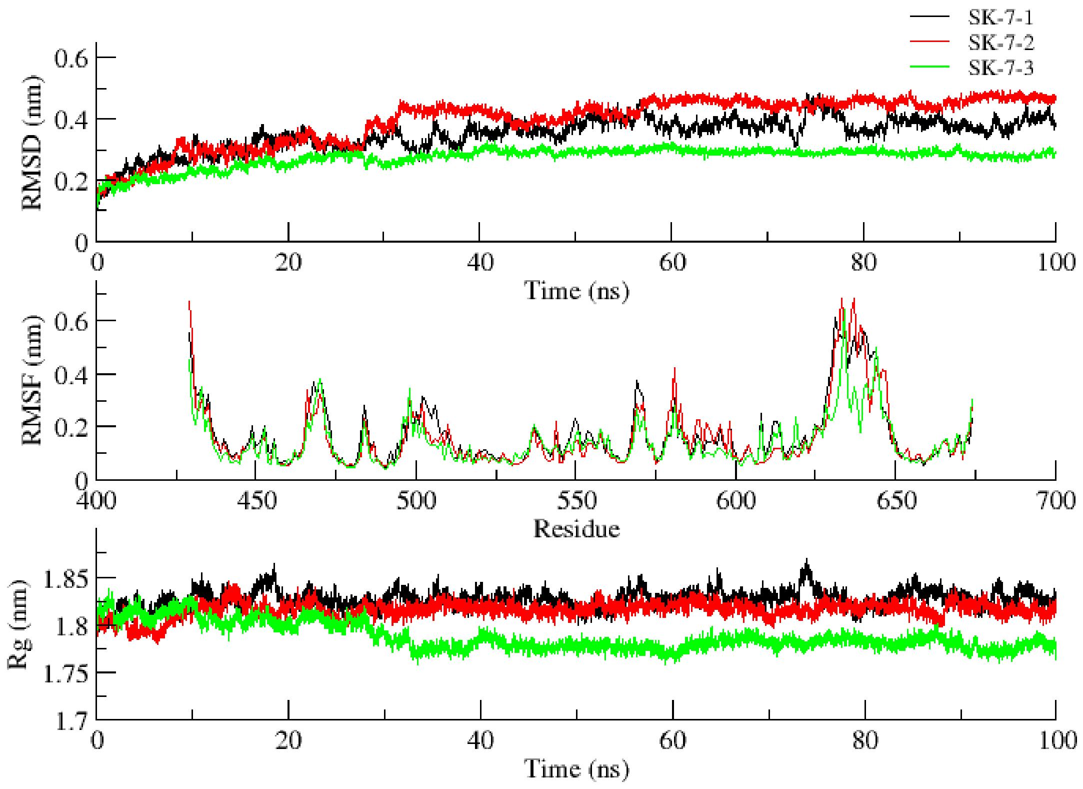

Figure S5. Root mean square deviation, root mean square fluctuation and radius of gyration of three replicas of 100ns MD simulation for pUL89/SK-7 complex

Table S1. Comprehensive detail of selected virtual hits against HCMV including chemical structures and results of docking studies.

Code	Structure	Database ID	Binding Affinity (kcal/mol)	Tanimoto Similarity	Interacted Residues
SK-1	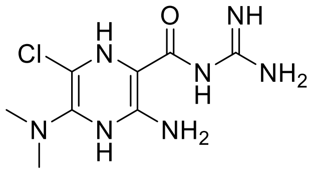	ChEMBL465179	-6.10	0.76	Asn469, Ser473, Glu534, Asn536, Thr537, Met579, Lys583
SK-2	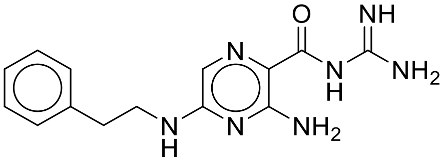	ChEMBL1962850	-6.15	0.77	Phe466 Asn469, Ser473, Asn536, Thr537, Met579, Asp650
SK-3	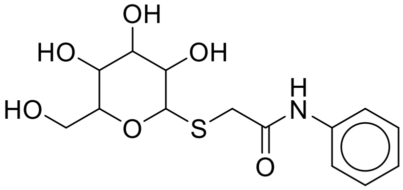	ChEMBL3351135	-5.39	0.79	Pro464, Asn469, Ser473, Asn536, Met579, Lys583
SK-4	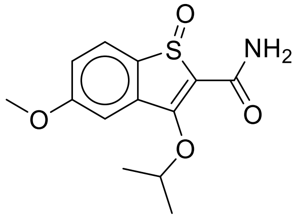	ChEMBL143702	-5.96	0.83	Phe466, Asn469, Ser473, Asn536
SK-5	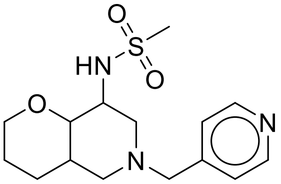	ChEMBL3553471	-6.45	0.80	Asn469, Ser473, Asn536, Lys583
SK-6	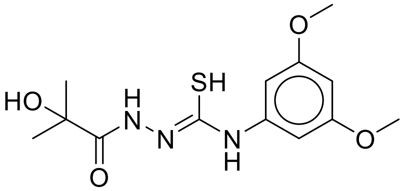	ChEMBL1360085	-6.23	0.75	Asn469, Ser473, Met579
SK-7	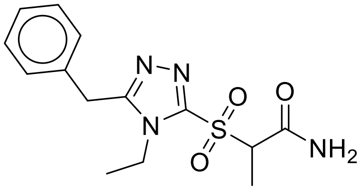	ZINC79951807	-6.33	0.75	Ser473, Asn536, Thr537, Met579, Lys583, Asp650
Cognate Ligand (2)	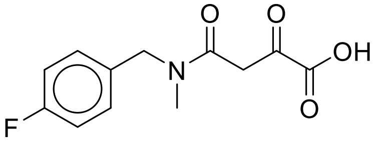	-	-5.31	1.0	Ser473, Phe466, Asn536, Thr537, Met579
